# Study of Atmospheric Pressure Plasma Temperature Based on Silicon Carbide Etching

**DOI:** 10.3390/mi14050992

**Published:** 2023-05-02

**Authors:** Shaozhen Xu, Julong Yuan, Jianxing Zhou, Kun Cheng, Hezhong Gan

**Affiliations:** College of Mechanical Engineering, Zhejiang University of Technology, Hangzhou 310023, China; 2112002412@zjut.edu.cn (S.X.);

**Keywords:** plasma temperature, silicon carbide, plasma etching, removal function

## Abstract

In order to further understand the excitation process of inductively coupled plasma (ICP) and improve the etching efficiency of silicon carbide (SiC), the effect of temperature and atmospheric pressure on plasma etching of silicon carbide was investigated. Based on the infrared temperature measurement method, the temperature of the plasma reaction region was measured. The single factor method was used to study the effect of the working gas flow rate and the RF power on the plasma region temperature. Fixed-point processing of SiC wafers analyzes the effect of plasma region temperature on the etching rate. The experimental results showed that the plasma temperature increased with increasing Ar gas until it reached the maximum value at 15 slm and decreased with increasing flow rate; the plasma temperature increased with a CF_4_ flow rate from 0 to 45 sccm until the temperature stabilized when the flow rate reached 45 sccm. The higher the RF power, the higher the plasma region’s temperature. The higher the plasma region temperature, the faster the etching rate and the more pronounced the effect on the non-linear effect of the removal function. Therefore, it can be determined that for ICP processing-based chemical reactions, the increase in plasma reaction region temperature leads to a faster SiC etching rate. By processing the dwell time in sections, the nonlinear effect caused by the heat accumulation on the component surface is effectively improved.

## 1. Introduction

Silicon carbide (SiC), one of the popular third-generation semiconductor materials, has excellent properties, such as a wide bandgap, a high critical breakdown electric field, and high thermal conductivity [1]. It is widely used in 5G communication, aerospace, and semiconductor lighting [2,3]. Due to the covalent bonding between Si and C atoms and the ordered, tightly packed structure, SiC has extremely high hardness (Mohs hardness 9.2) and chemical inertness, which makes SiC a typical hard-to-process material [4,5]. The current SiC ultra-precision machining techniques mainly include electrochemical polishing (ECMP), chemical mechanical polishing (CMP), and magnetorheological processing (MRF). In the above technology, CMP is the only effective means to achieve both surface planarization and global polishing, but the polishing efficiency is generally low. ECMP adds an electric field based on CMP to improve the material removal rate and can process arbitrarily shaped workpieces, but its processing cost is high, and the polishing liquid has the possibility of chemical pollution. MRF has the advantages of a good removal effect and strong controllability. However, the properties of magnetorheological fluid are easily affected by external factors, such as temperature and viscosity; the removal effect is unstable; and the high cost of MRF devices makes it difficult for practical application in industrial fields [6,7,8,9,10]. With the growing demand for processing SiC materials, atmospheric pressure plasma processing methods are gaining more and more attention.

According to the different methods of plasma excitation generation, atmospheric plasma can be divided into inductively coupled plasma (ICP) and capacitively coupled plasma (CCP) [11,12]. Among the institutions that have conducted plasma research, it is mainly Osaka University in Japan and the Institute of Surface Modification (IOM) in Germany that used CCP to generate plasma. Osaka University adopted various forms of electrodes for different machining requirements, which were named plasma chemical vaporization machining (PCVM) [13]. IOM had inherited the technological advantage of ion beam processing by using a 2.45 GHz microwave plasma power source to generate excited plasma, called plasma jet machining (PJM) [14]. The Lawrence Livermore National Laboratory (LLNL) first proposed reactive atomic plasma (RAP) processing in 1999, which uses ICP to obtain plasma in a high gas pressure environment [15]. However, because the ICP will generate a large amount of joule heat, it is difficult to cool down the plasma inside the generator to ensure the safety of the process. To solve this problem, Cranfield University tried to minimize the negative effects caused by high temperatures during processing and found that improving the temperature by changing the flow rate of the reaction gas had a very limited effect [16]. The scholar Renaud Jourdain built a simulation model and confirmed that the nozzles have a significant cooling effect on the plasma jet [17]. However, the nozzles also bring a certain degree of influence to the process. In the study of RAP processing of ultra-low expansion coefficient glass after adding nozzles and fused silica glass, it was found that the volume removal of material from static fixed-point processing elements was not fully linear but rather had a distinct curvilinear relationship. They tentatively attributed the generation of this phenomenon to nonlinearity due to the effect of temperature on the chemical reactions produced [18]. Different from the above techniques, atmospheric pressure plasma polishing (APPP), proposed by the Harbin Institute of Technology, is characterized by the use of CCP for micro-trimming surface shape and ICP for fast and efficient removal [19].

In addition to the above concerns, during atmospheric plasma excitation, many factors, such as gas flow rate, directly affect the plasma region temperature, which influences the chemical reaction rate during material removal. Some scholars have studied the temperature variation of CCP [20], but the study of plasma region temperature for the ICP excitation process has not been published. Therefore, in this paper, based on the ICP excitation method, the following studies were conducted: firstly, we measured the temperature of the plasma reaction region by infrared thermometry and investigated the influence of the excitation gas Ar flow rate, reaction gas CF_4_ flow rate, and RF power magnitude on the plasma region temperature by the single-factor method. Then we studied the influence of plasma region temperature and dwell time on the SiC etching efficiency by using fixed-point etching of SiC material, which laid the foundation for the subsequent study of SiC planarization processing.

## 2. Materials and Methods

**Figure 1:**
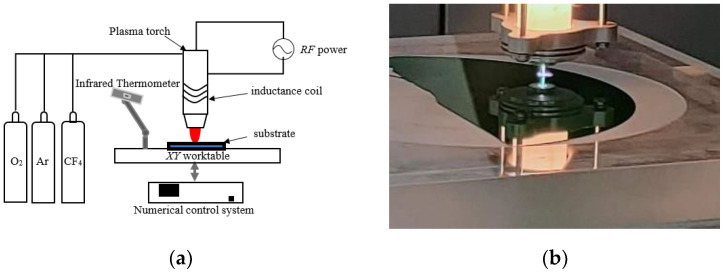
ICP processing system. (**a**) schematic diagram; (**b**) physical picture.

The ICP processing system mainly consists of five subsystems, including the mechanical motion control system, gas supply system, plasma-generating device, RF power supply, and external modules, as shown in Figure 1a.

The mechanical motion control system includes motion stages (e.g., *X*- and *Y*-axis tables) and a numerical control system that controls the motion of the plasma-generating device. The gas supply system provides two gases: one is the excitation gas, which is the main source of protective plasma and consists mainly of the rare gas Ar; the other is the reaction gas, which is the main source of reactive plasma and consists mainly of F-containing gas (CF_4_ or SF_6_) and O_2_. CF_4_ is used in the following experiments as the reaction gas. The external module is an infrared thermometer, which is responsible for measuring the temperature of the plasma reaction area. The outer layer of the plasma generator is connected to an inductive coil, and when the RF power supply is powered up, the tube ionizes the gas under the coupling effect of the electric field and the peripheral coil to produce reactive etching particles F*, which react with the substrate material to convert the solid substrate material into a gaseous compound. The expression of the reaction between F* and SiC material is as follows:

(1) $$\mathrm{SiC}+F^{*}+O\to \mathrm{Si}F_{4}↑+CO_{2}↑+\mathrm{CO}↑$$

In the above equation, SiC is the main material of the substrate; the active particles F* and O are formed by ionization of CF_4_ and O_2_, respectively; and the products SiF_4_, CO_2_, and CO are volatile gases. A physical picture of the processing system is shown in Figure 1b.

**Figure 2:**
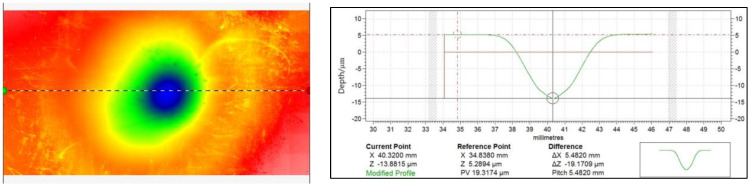
APPP removal contour.

The atmospheric plasma removal function is similar to conventional ion beam machining in that it has a Gaussian shape. The removal profile cross-section after fixed-point processing, which uses common processing parameters, is shown in Figure 2. The APPP removal profile is generally fitted with a standard Gaussian function with the following expression:

(2) $$R\left(x\right)=a*e^{\frac{x^{2}+y^{2}}{2\sigma^{2}}}$$

(3) $$FWHM=2\sqrt{2\ln2\sigma }$$

In the above formula, a is the removal depth, that is, the peak of the removal function; the peak of the removal function per unit time can also be expressed as the removal rate. FWHM indicates the size of the half-height width of the removal function, that is, the width value in the horizontal direction when the removal depth is half of the peak; σ is the standard deviation of the Gaussian function, determined by the half-height width of the removal function, FWHM, as shown in Equation (3) above.

## 3. Temperature Measurement Method

**Figure 3:**
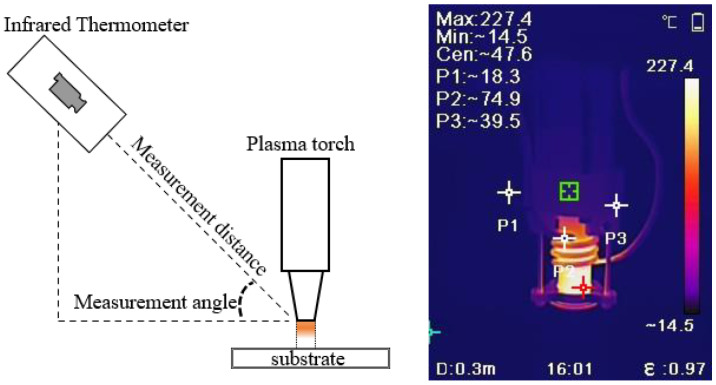
Schematic diagram and effect drawing of the infrared thermometer.

The most commonly used methods for plasma region temperature measurement are the spectral intensity method, the probe method, and the infrared thermometry method [21]. The principle of the spectral intensity method is to use a spectrometer to measure the absolute intensity of different spectra emitted by the plasma, combined with an analysis of the spectral emission theory to obtain the plasma’s internal temperature. However, since most spectrometers still measure the relative intensity of the emission spectrum rather than the absolute intensity, experimental spectral thermometry research for measuring atmospheric plasma is not yet complete, and the probe method is not suitable for an atmospheric pressure environment. In contrast, infrared thermometry has many advantages, such as speed, lightness, and safety, which have made the use of infrared thermometry increasingly popular in various fields [22]. Therefore, in this paper, infrared thermometry is chosen to measure the plasma region’s temperature. Erroneous use of infrared thermometers may cause significant errors in the measured temperature. Therefore, the temperature measurement experiment follows these specifications: (1) fix the infrared thermometer to keep the measuring distance and the measuring angle constant; (2) select the correct material emissivity; (3) after adjusting the experimental parameters, wait for a certain time to measure after the plasma region temperature is stable. Figure 3 shows the diagram of the infrared thermometer and the infrared radiation of the plasma torch.

## 4. Results and Discussion

### 4.1. Effect of Processing Parameters on Plasma Region Temperature

#### 4.1.1. Effect of Ar Gas Flow Rate

Ar gas, as one of the most common inert gases, does not form stable compounds with other elements at room temperature, so it is mainly used as an excitation gas or protective gas in ICP excitation. After the RF power supply is charged, the Ar molecules are continuously hit by high-energy electrons in the strong electromagnetic field to obtain internal and kinetic energy, and the particles are transformed from the ground state to the high-energy state. This refers to active Ar atoms, which are mixed with high-energy electrons and a variety of ground-state particles in the field to form an electrically neutral Ar plasma. If Ar gas is the main source of plasma generation and its flow rate is too low or too high, the formed plasma flame will be unstable or even extinguished, so the Ar gas flow range is generally around 11–27 slm. This section sets the experimental parameters under different Ar flow rates, as shown in Table 1.

**Figure 4:**
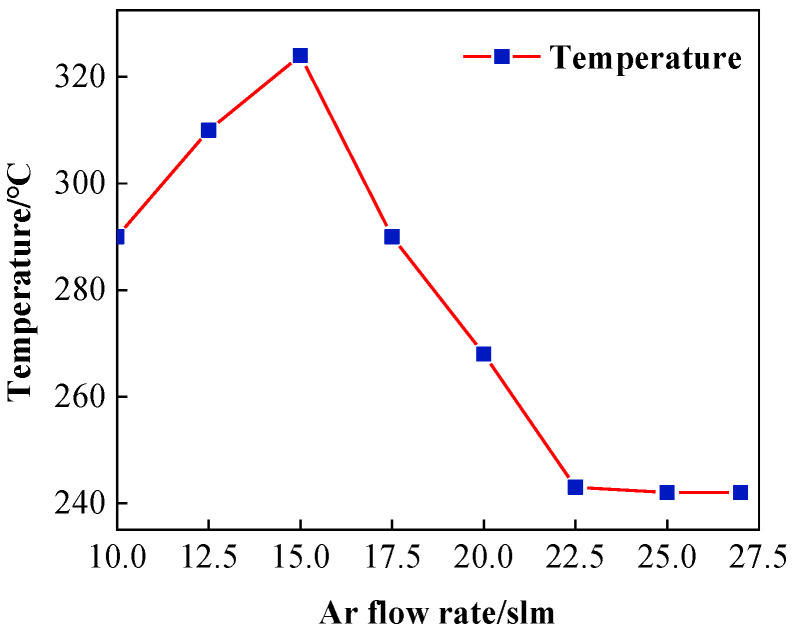
Influence of Ar flow rate on plasma temperature.

The experimental results are shown in Figure 4. The plasma region temperature keeps rising when the Ar flow rate is between 10 and 15 slm, then decreases instead of rising as the Ar gas flow rate increases, which is due to the limited ionization capacity of the RF power of 500 W. The ionization capacity is saturated when the Ar gas flow rate reaches 15 slm, at which time there are no more free electrons generated in the electromagnetic field and no more heat can be released. The excess Ar gas is passed through the plasma field in the form of low-energy molecules flowing from the nozzle to the air, which plays the role of heat dissipation, so the temperature of the plasma decreases. Additionally, the molecular form of Ar inhibits the generation of active reaction particles, thus making the etching efficiency decrease. Excess Ar gas also makes the plasma flame formed from the nozzle more elongated, so the shape of the removal profile of the workpiece also changes, and the Ar gas flow rate generally cannot be too low.

#### 4.1.2. Effect of CF_4_ Gas Flow Rates

When CF_4_ is the reaction gas, the addition of a small amount of O_2_ gas serves to enhance the F* etching efficiency and inhibit deposit generation [23,24], and its excitation principle is similar to that of CF_4_ gas. Therefore, this paper only focuses on the effect of the CF_4_ gas flow rate on the plasma region temperature. After CF_4_ is passed into the generator, it undergoes multi-stage decomposition under the influence of the active Ar plasma atmosphere and the internal electromagnetic field. Active F-atoms are generated, and this particle interacts with the substrate surface to achieve removal. Considering the completeness of the experiment and the fact that this section only deals with the effect of the gas flow rate on the plasma region temperature, CF_4_ gas flow rates of 0 sccm and 5 sccm were added for comparison, as shown in Table 2.

**Figure 5:**
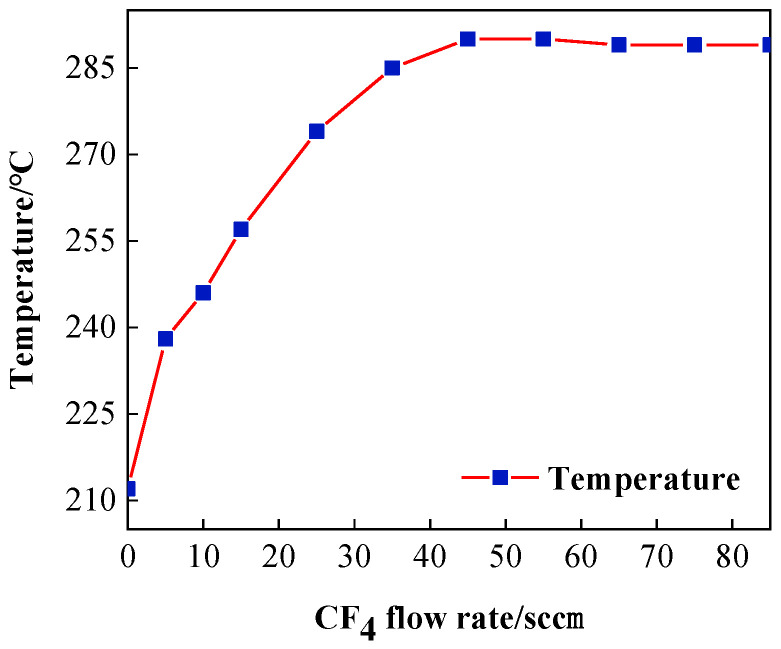
Influence of CF_4_ gas flow on plasma temperature.

The plasma region temperature change curve with CF_4_ flow is shown in Figure 5. When the CF_4_ flow range is around 0–45 sccm, a large number of free electron collisions constantly enter the F atom and get enough energy after being excited by high-energy F ions to lead to a sharp rise in the plasma torch temperature, and at this time the plasma flame at the nozzle will become light green. This is because in the high-energy state, active F atoms are not stable, and a large number of photons are released in the process of returning to the ground state. When the flow rate of CF_4_ is greater than 45 sccm, after the RF electric field excitation capacity reaches saturation, the excitation ratio of F atoms in the generator remains unchanged. The excess CF_4_ gas flows out in its original form, so the temperature of the plasma torch tube gradually flattens out with the increase in CF_4_ gas flow rate and even slightly decreases. This is the same reason why the temperature drops when the Ar gas flow rate is excessive. However, because the CF_4_ gas flow rate increases by only 10 sccm, which is too small compared to the proportion of Ar gas in the tube, the cooling trend of the plasma is less obvious.

#### 4.1.3. Effect of RF Power

The RF power supply is the energy input source for the plasma processing system. RF input power indicates the energy input to the plasma torch and is an important parameter of plasma excitation. The size of the RF input power determines whether it can discharge and the size of the discharge intensity. When the electric field strength is greater, the electron concentration increases, a large number of electrons hit the reaction gas, dissociate more active etching particles, and the APPP etching rate increases. Theoretically, the higher the RF input power, the higher the Ar gas excitation intensity, and the temperature will increase [25]. This section sets the parameters under different RF power values, as shown in Table 3, to experimentally prove that the reasonable RF input power range based on this parameter is around 350–700 W. Too high or too low will produce an arc-pulling phenomenon or even quenching. 

**Figure 6:**
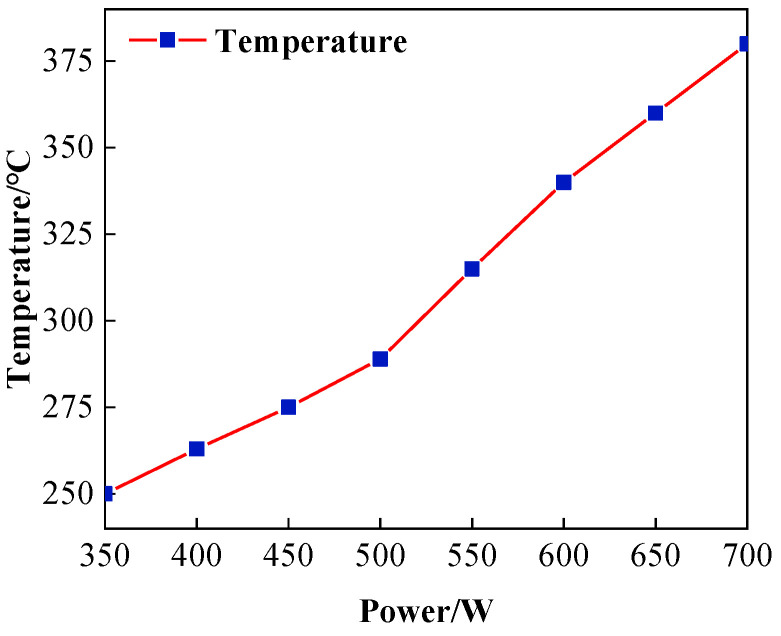
Influence of RF power on plasma temperature.

Figure 6 shows the change curve of the plasma region temperature at different RF power, the RF power from 350 W to 700 W plasma region temperature steadily increases, which is also in line with the above theory. The acceleration of temperature rise increases when 600 W, so the RF power should be controlled below 600 W in practical processing.

### 4.2. Study on the Removal Function of Silicon Carbide by ICP Processing

#### 4.2.1. Effect of Plasma Region Temperature on the Removal Function

Atmospheric plasma processing is not affected by the physical properties of the material, so it can perform efficient and rapid processing of the material, the essence of which is material removal through the chemical reaction between the active particles generated under the active atmosphere of the plasma and the surface of the substrate. Its reaction rate is also in accordance with the Arrhenius formula with the following expressions [26,27]:

(4) $$k=Ae^{-\frac{E_{a}}{RT}}$$

In the above equation, *k* is the chemical reaction rate constant; *A* is the Arrhenius constant; *E_a_* is the reactivity energy, in units (J/mol); *T* is the absolute temperature, in units K; and *R* is the molar gas constant, in units (J/mol-K). The etching efficiency of ICP-processed silicon carbide varies with the temperature of the plasma reaction region. In order to study the effect of plasma region temperature on silicon carbide under the same process parameters, in this section, the experimental parameters of the conventional processing were made constant. The processing distance between the nozzle tip and the sample surface was 4 mm, the dwell time was 30 s, the Ar flow rate was 19 slm, the CF_4_ flow rate was 60 sccm, the O_2_ flow rate was 10 sccm, and the RF power supply power was 500 W. It should be noted that the workpiece must be completely cooled at intervals to prevent thermal accumulation. The experimental results were obtained by preheating to get the plasma reaction area temperatures of 256 °C, 288 °C, and 297 °C for three groups of fixed-point etching, which were recorded as points A, B, and C, respectively.

**Figure 7:**
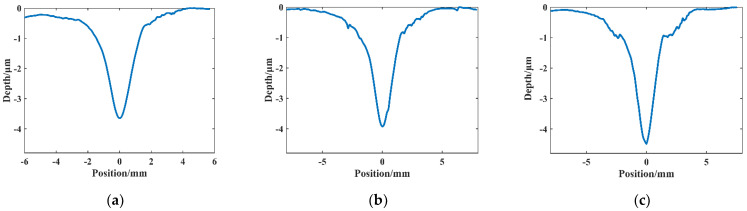
Cross-sectional profile of the removal function at different plasma temperatures: (**a**) point A, 256 °C; (**b**) point B, 288 °C; and (**c**) point C, 297 °C.

The profile data of the machined section at three points were measured using a Taylor profiler and imported into Matlab to obtain Figure 7. The peak removal depths were 3.64 μm, 3.93 μm, and 4.47 μm at points A, B, and C, respectively. The increase in longitudinal etching caused the peak removal profile to increase with temperature, indicating that the increase in plasma region temperature promotes the etching rate of the active particles on the SiC material. Hence, the higher the plasma region temperature, the higher the removal depth peak for the same dwell time. Comparing the temperature change, it is found that when the temperature increases from 256 °C to 288 °C, the peak removal depth increases by 0.29 μm, with a ratio of about 0.009 μm/°C to the temperature increment, while from 288 °C to 297 °C, the peak removal depth increases by 0.54 μm/min, with a ratio of about 0.06 μm/°C to the temperature increment. This is much larger than the first two points of difference ratio change, which indicates that the influence of the plasma area temperature on the etching efficiency is more significant with the increase in temperature. Therefore, in the actual processing, it is necessary to ensure the stability of the processing environment while controlling the plasma temperature within a small range to ensure the stability of the plasma removal capacity.

#### 4.2.2. Effect of Dwell Time on the Removal Function

**Figure 8:**
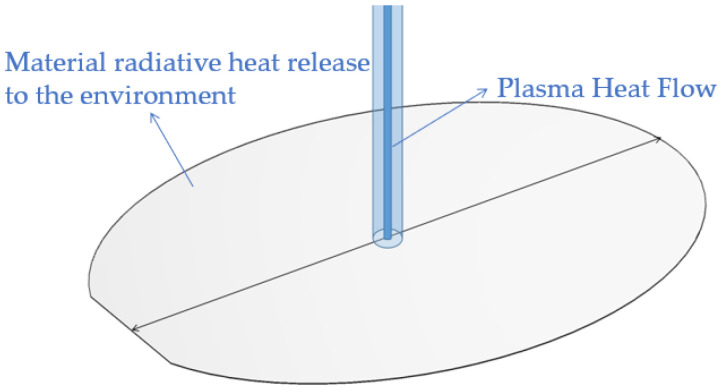
Plasma thermal radiation schematic.

In ICP fixed-point or planar processing, the plasma flame equivalent to a fixed-point heat source will continuously conduct heat transfer to the substrate surface to increase its temperature, as shown in Figure 8, so the relationship between the dwell time and etching rate will not be a simple linear fit [28].

To investigate the effect of heat removal due to increasing dwell time, the dwell time is set in this section, as shown in Table 4, and other parameters are the same as in the previous section. In addition to this, the same five processing points were set without changing other parameters, and their dwell times were divided into 10-s increments, as shown in Table 4. For example, the processing point with a dwell time of 6 × 10 in the table means that the point was processed six times for 10 s each time. Sufficient intervals were made between each processing so that the surface of the SiC wafer processed twice was sufficiently cooled to minimize the temperature accumulation on the wafer surface. To facilitate the distinction between the two types of processing, the processing point with uninterrupted dwell time is recorded as a single processing point, and the interval division is recorded as multiple processing.

**Figure 9:**
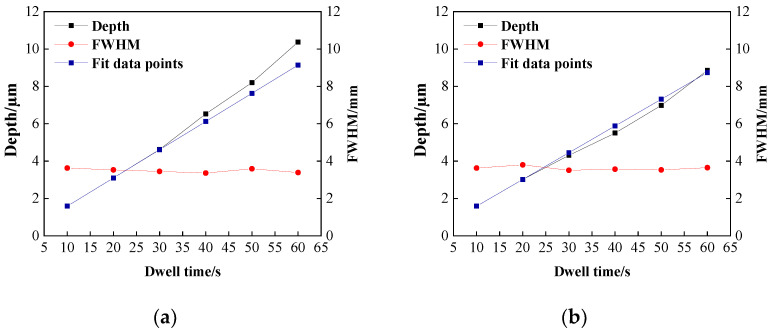
Removal function and dwell time variation curve: (**a**) single processing; (**b**) multiple processing.

The experimental results are shown in Figure 9. Figure 9a shows the results of single processing, and Figure 9b shows the results of multiple processing. The blue curve in the figure represents a simple linear fit to the removal depths of the first two processes to compare the extent to which the experimentally obtained removal depth curves deviate from linearity. Theoretically, when the dwell time of a section is constant, then the total dwell time of the same removal amount should be equal. However, when comparing the peak depth of removal curves of single processing and multiple interval processing, it can be found that the greater the dwell time, the greater the deviation from the linear curve of single processing under the same cumulative dwell time of both. This is consistent with the conclusion about the effect of temperature on the depth of removal in the previous section. This indicates that the thermal accumulation on the wafer surface is the reason for the non-linear relationship between the dwell time and etching rate, and the longer the dwell time accumulation, the greater the effect on the instability of the etching rate. Therefore, in the planarization processing, the temperature accumulation on the wafer surface can be reduced by speeding up the process multiple times, and at the same time, the temperature accumulation on the wafer surface can be dissipated as much as possible.

## 5. Conclusions

In this paper, based on ICP processing of SiC material, the temperature variation in atmospheric plasma processing was measured by infrared thermometry, and the influence of the three main processing parameters, Ar, CF_4_, and RF power, on the temperature in the plasma region was analyzed by the single-factor method. The following conclusions were drawn in combination with the profilometer measurements:

(1) In atmospheric plasma processing, plasma temperature fluctuations affect the working gas excitation, the decomposition process, and the energy and distribution of active particles on the surface of the components, which further affect the removal stability of the material. Infrared thermometry reveals that the plasma region temperature rises with increasing Ar gas flow rate, reaching a maximum of 325 °C at 15 slm. With the reaction gas CF_4_ flow rate in the interval of around 0–45 sccm, the plasma will be continuously exothermic with the ionization of the system’s overall temperature increase until the flow rate reaches 45 sccm, at which point the system’s ionization capacity reaches saturation after the temperature stabilizes with only a small decline. The size of the RF input power determines the size of the discharge and its intensity. When the RF power is around 350–700 W, the plasma region temperature increases with the increase in RF power.
(2) The higher the plasma region temperature under the same parameters, the faster the etching rate of ICP-etched SiC, and the higher the plasma region temperature, the more easily the removal rate is affected. Additionally, the dwell time has a non-linear relationship with the peak removal depth due to the effect of heat accumulation on the wafer surface.

## Figures and Tables

**Table 1:** Processing parameters under different Ar flow rates.

Parameter	Value
Ar flow rate/slm	10, 12.5, 15, 17.5, 20, 22.5, 25, 27
CF_4_ flow rate/sccm	60
O_2_ flow rate/sccm	10
Input power/W	500

**Table 2:** Processing parameters under different CF_4_ flow rates.

Parameter	Value
Ar flow rate/slm	19
CF_4_ flow rate/sccm	0, 5, 10, 15, 25, 35, 45, 55, 65, 75, 85
O_2_ flow rate/sccm	10
Input power/W	500

**Table 3:** Processing parameters under different RF power values.

Parameter	Value
Ar flow rate/slm	19
CF_4_ flow rate/sccm	60
O_2_ flow rate/sccm	10
Input power/W	350, 400, 450, 500, 550, 600, 650, 700

**Table 4:** Experimental parameters with different dwell times.

Processing Method	Dwell Time/s
Single process	10, 20, 30, 40, 50, 60
Multiple process	10 × 1, 10 × 2, 10 × 3, 10 × 4, 10 × 5, 10 × 6

## References

[1] Nguyen T.K., Aberoumand S., Dao D.V. (2021). Advances in Si and SiC materials for high-performance supercapacitors toward integrated energy storage systems. Small.

[2] Kimoto T. (2015). Material science and device physics in SiC technology for high-voltage power devices. Jpn. J. Appl. Phys..

[3] Pearton S.J., Yang J.C., Cary P.H., Ren F., Kim K., Marko J., Tadjer M.K., Mastro M.A. (2018). A review of Ga_2_O_3_ materials, processing, and devices. Appl. Phys. Rev..

[4] Kovalcikova A., Sedlacekb J., Lencesb Z., Bystrickyb R., Duszaa J., Sajgalikb P. (2016). Oxidation resistance of SiC ceramics prepared by different processing routes. J. Eur. Ceram. Soc..

[5] Seo Y.K., Kim Y.W., Nishimura T., Seoc W.S. (2016). High-temperature strength of a thermally conductive silicon carbide ceramic sintered with yttria and Scandia. J. Eur. Ceram. Soc..

[6] Luo Y.G., Xiong Q., Lu J.B., Yan Q.B., Hu D. (2022). Chemical mechanical polishing exploiting metal electrochemical corrosion of single-crystal SiC. Mater. Sci. Semicond. Process..

[7] Adia H., Doi T., Takeda H., Katakura H., Kim S.W., Koyama K., Yamazakib T., Unedab M. (2012). Ultraprecision CMP for sapphire, GaN, and SiC for advanced optoelectronics materials. Curr. Appl. Phys..

[8] Deng H., Hosoya K., Imanishi Y., Endo K., Yanmamura K. (2015). Electro-chemical mechanical polishing of single-crystal SiC using CeO_2_, slurry. Electrochem. Commun..

[9] Alam Z., Khan D.A., Jha S. (2018). A study on the effect of polishing fluid volume in ball end magnetorheological finishing process. Mater. Manuf. Process..

[10] Liang H.Z., Yan Q.S., Lu J.B., Gao W.Q. (2016). Experiment on Chemical Magnetorheological Finishing of SiC Single Crystal Wafer. Mater. Sci. Forum.

[11] Verma Y., Chang A.K., Berrett J.W., Futtere K., Gardopee G.J., Kelley J., Kyler T., Lee J., Lyford N., Proscia D. (2006). Rapid damage-free shaping of silicon carbide using reactive atom plasma(RAP) processing. SPIE Astronomical Telescopes Instrumentation.

[12] Piechulla P., Bauer J., Boehm G., Paetzelt H., Arnold T. (2016). Etch mechanism and temperature regimes of an atmospheric pressure chlorine-based plasma jet process. Plasma Process Polym.

[13] Yamamura K., Shimada S. (2008). Damage-free Improvement of Thickness Uniformity of Quartz Crystal Wafer by Plasma Chemical Vaporization Machining. CIRP Ann. Manuf. Technol..

[14] Arnold T., Boehm G., Eichentopf I.M. (2010). Plasma Jet Machining. J. Vac. Sci. Technol..

[15] Fiske P.S., Verma Y., Chang A., Lyford N., Kelley J., Sommer P., Li N., Pang K., Gardopee G., Kyler T. (2006). Reactive atom plasma processing for lightweight SiC mirrors. OSA Technical Digest.

[16] Castelli M., Jourdain R., Morantz P., Shorea P. Fast figuring of large optics by reactive atom plasma. Proceedings of the SPIE 8450.

[17] Yu N., Jourdain R., Gourma M., Shorea P. (2016). Analysis of De-Laval nozzle designs employed for plasma figuring of surfaces. Int. J. Adv. Manuf. Technol..

[18] Castelli M., Jourdain R., McMeeking G., Morantz P., Shore P., Proscia D., Subrahmanyan P. Initial strategies for 3D RAP processing of optical surfaces based on a temperature adaptation approach. Proceedings of the 36th International MATADOR Conference.

[19] Wang D.F., Jin H.L., Jin J., Bo W. (2011). Research on the Influence of Reactive Gas CF_4_/SF_6_ upon the Temperature of Atmospheric Pressure Plasma Jet. Aviat. Precis. Manuf. Technol..

[20] Zhao X., Jin H.L., Li N., Jiang J., Bo W. (2012). Research on Influence of Plasma Gas He and process Time upon Temperature of Atmospheric Pressure Plasma Jet. Aviat. Precis. Manuf. Technol..

[21] Lin Q., Ren Q.L. (2005). Investigation in Radiation Characteristic of Atmospheric Pressure Glow Discharge Plasma in Air. J. Xiamen Univ. (Nat. Sci.).

[22] Shen X., Deng H., Zhang X., Peng K., Yamamura K. (2016). Preliminary study on atmospheric-pressure plasma-based chemical dry figuring and finishing of reaction-sintered silicon carbide. Opt. Eng..

[23] Yan Y.Y., Chan-Park M.B., Yue C.Y. (2005). CF_4_ plasma treatment of poly(dimethylsiloxane): Effect of fillers and its application to high-aspec-ratio UV embossing. Langmuir.

[24] Wang D.F. (2013). Research on atmospheric pressure plasma processing technology of fused silicon. J. Shaanxi Univ. Technol. (Nat. Sci. Ed.).

[25] Su X. (2016). Design and Study on the Atmospheric Pressure Plasma Jet Torch. Master’s Thesis.

[26] Laidler K.J. (1984). The development of the Arrhenius equation. J. Chem. Educ..

[27] Tang W.J., Liu Y.W., Zhang H. (2003). New approximate formula for Arrhenius temperatureintegral. Thermochim. Acta.

[28] Peng B., Dun A.H., Wu L.Z., Wang Z., Xu X.K. (2021). Variable Removal Function in Atmospheric Pressure Plasma Polishing. Chin. J. Lasers.

